# Management of Childhood Obesity—Time to Shift from Generalized to Personalized Intervention Strategies

**DOI:** 10.3390/nu13041200

**Published:** 2021-04-06

**Authors:** Mohamad Motevalli, Clemens Drenowatz, Derrick R. Tanous, Naim Akhtar Khan, Katharina Wirnitzer

**Affiliations:** 1 Department of Sport Science, Leopold-Franzens University of Innsbruck, A-6020 Innsbruck, Austria; derrick.tanous@student.uibk.ac.at (D.R.T.); katharina.wirnitzer@ph-tirol.ac.at (K.W.); 2 Division of Sport, Physical Activity and Health, University College of Teacher Education Upper Austria, A-4020 Linz, Austria; clemens.drenowatz@ph-ooe.at; 3 Nutritional Physiology & Toxicology Division, INSERM UMR 1231, Université de Bourgogne, F-21000 Dijon, France; naim.khan@u-bourgogne.fr; 4 Department of Subject Didactics and Educational Research & Development, University College of Teacher Education Tyrol, A-6020 Innsbruck, Austria; 5 Life and Health Science Cluster Tirol, Subcluster Health/Medicine/Psychology, A-6020 Innsbruck, Austria; 6 Research Center Medical Humanities, Leopold-Franzens University of Innsbruck, A-6020 Innsbruck, Austria

**Keywords:** children, adolescents, overweight, obesity, weight management, lifestyle, body composition

## Abstract

As a major public health concern, childhood obesity is a multifaceted and multilevel metabolic disorder influenced by genetic and behavioral aspects. While genetic risk factors contribute to and interact with the onset and development of excess body weight, available evidence indicates that several modifiable obesogenic behaviors play a crucial role in the etiology of childhood obesity. Although a variety of systematic reviews and meta-analyses have reported the effectiveness of several interventions in community-based, school-based, and home-based programs regarding childhood obesity, the prevalence of children with excess body weight remains high. Additionally, researchers and pediatric clinicians are often encountering several challenges and the characteristics of an optimal weight management strategy remain controversial. Strategies involving a combination of physical activity, nutritional, and educational interventions are likely to yield better outcomes compared to single-component strategies but various prohibitory limitations have been reported in practice. This review seeks to (i) provide a brief overview of the current preventative and therapeutic approaches towards childhood obesity, (ii) discuss the complexity and limitations of research in the childhood obesity area, and (iii) suggest an Etiology-Based Personalized Intervention Strategy Targeting Childhood Obesity (EPISTCO). This purposeful approach includes prioritized nutritional, educational, behavioral, and physical activity intervention strategies directly based on the etiology of obesity and interpretation of individual characteristics.

## 1. Introduction

The examination of the etiology of childhood obesity is a growing area of research aiming to yield important insights for public health [1,2]. During the last three decades, the annual growth rate of publications on childhood obesity (average of 11.6% per year) has been generally higher than other sub-areas in the pediatric field and biomedical research [3]. Given the rising prevalence of childhood obesity in most developed and developing countries, it is now considered a global pandemic [4]. Worldwide, an estimated 170 million children are considered overweight or obese currently [5], and approximately more than half of them are predicted to become obese adults [6].

These trends in excess body weight may also contribute to an increase in chronic cardio-metabolic disorders, typically observed only in adults (e.g., hypertension, hyperglycemia, and dyslipidemia), but are becoming increasingly common in children and adolescents with obesity [7]. Additionally, pediatric populations with obesity are known to have several psychosocial problems including discrimination, social isolation, and low self-esteem, which affect their health, education, and quality of life [6,8]. Furthermore, the crosstalk between obesity and many viral pandemics, such as the 2009 swine flu [9] or the current COVID-19 pandemic [10,11], has provided new insights into mortal characteristics of this chronic syndrome.

The etiology of obesity is complicated and multifactorial, which indicates that excess body weight results from a complex interaction of a broad range of factors [12]. In addition to genetic vulnerability as a well-recognized internal factor contributing to excess body weight, a wide range of physiological disorders, as well as modifiable environmental factors and obesogenic behaviors, play key roles in the development of obesity [11,13,14]. Amongst children, the most common obesogenic behavior includes high consumption of unhealthy foods, low levels of physical activity (PA), high levels of mental stress, high levels of screen time, and poor sleep patterns [1,2,15]. These behaviors are influenced by several factors and interactions involving genetics, interpersonal relationships, and the environment [16,17]. Additionally, some evidence indicates that obesity-related behaviors are highly context-dependent and are influenced by several biopsychosocial factors [18]. When discussing the gene–environment interplay in the etiology of obesity, it is believed that the contribution to an obese phenotype is not “nature or nurture”, but rather “nature via nurture” [12]. Recently, Jackson et al. emphasized in their review that biology plays a fundamental role in determining the amount of body fat in addition to environmental factors [12]. Moreover, genetically predetermined obesogenic behavior seems to have a significant relationship with environmental influence on body weight [12], which could add to the complexity of obesity. This complex interaction is exemplified further by the role of energy flux (the rate of energy expenditure and energy intake) in the regulation of energy balance [19]. The complicated nature of childhood obesity which leads to a wide range of inter-individual differences highlights the importance of child-centered specific approaches, particularly personalized interventions, for managing childhood obesity.

Current scientific insights are limited in successfully decelerating the rise of the childhood obesity pandemic; the present review’s primary goal is to establish a novel translational link between the literature and practice by introducing applied strategies targeting childhood obesity. Therefore, the purpose of this narrative review is to provide a brief overview of the current preventative and therapeutic approaches towards the management of childhood obesity (focusing on their limitations and complexity), and accordingly, to suggest an Etiology-Based Personalized Intervention Strategy Targeting Childhood Obesity (EPISTCO) as a purposeful approach to prioritize and implement nutritional, PA, and lifestyle intervention strategies based on the etiology of obesity and interpretation of individual characteristics. These objectives are based on the limited success of previous efforts targeting childhood obesity.

## 2. Weight-Related Behaviors in Children

Comprehensive clinical guidelines and recommendations to diagnose, prevent, and treat childhood obesity have been well documented for pediatric specialists to implement at different stages in obesity prevention and treatment programs [20,21].

Nutrition and dietary pattern not only affect anthropometry and body composition in children and adolescents, but they also influence neurocognitive and psychomotor development [22,23]. Evidence indicates that when compared to adults, children and adolescents are at a higher risk of insufficient intake of certain food groups (e.g., whole grains and/or unprocessed foods) [7,24], which may contribute to the increased risk of obesity [7]. Calorically restricted diets are widely used to target childhood obesity [22], but potentially contribute to nutrient deficiencies, which may impair growth and development [22,25]. The importance of adequate nutrient consumption for growth and development also emphasizes the need for targeting energy expenditure by ensuring sufficient PA. Even though detrimental effects of insufficient PA on various health and weight outcomes are well-documented, results from a comprehensive analysis including 1.6 million adolescents from a pooled number of 298 school-based studies from 146 countries show that 80% of adolescents do not meet PA recommendations of at least 60 min of PA with moderate to vigorous intensity over 5 d/w, which puts their current and future health at risk [26].

In addition to poor nutrition and low PA, results from different studies show that most children and adolescents do not meet obesity-associated lifestyle guidelines, such as recommendations for sleep and screen time [27,28]. According to the Centers for Disease Control and Prevention (CDC), 60% to 70% of the American pediatric population does not meet the American Academy of Pediatrics (AAP) sleep recommendations [1,27] of 10–13 h for 3- to 5-year-olds, 9–12 h for 6- to 12-year-olds, and 8–10 h for 13- to 18-year-olds [29]. Short duration and late sleep timing can contribute to the onset and development of childhood obesity [30], particularly by altering appetite-regulating hormones and consequential eating disorders [31]. Parents can play an important role in fostering healthy sleep patterns by arranging sleep time, providing a calm atmosphere, and keeping screens away before bedtime [32]. Higher amounts of daily screen time contribute to obesity due to their association with a reduced feeling of satiety, increased consumption of unhealthy and energy-dense snacks [33], and poor sleep patterns [34]. The AAP recommends that daily nonacademic screen time (TV, video games, and mobile phone) should not exceed one hour for 2- to 5-year-olds, and two hours for ≥6-year-old children, and there should be parental supervision of content watched [1]. Available data, however, indicates that the majority of children has an extraordinarily high daily screen time [28,35], up to an average of 6 h/day among 13- to 18-year-olds [36] and 7 h/day among 8- to 18-year-olds [37] when TV, computer, mobile devices, and web-based sources are combined. Sedentary behaviors, screen time, and sleep abnormalities can be even higher during annual vacation due to the absence of a regular schedule [38].

## 3. Strategies for the Prevention and Treatment of Childhood Obesity

To date, the safety and efficacy of various approaches on the management of childhood obesity have been reported by numerous experimental and cross-sectional studies as well as reviews and meta-analyses [2,6,15,39,40]. Nevertheless, pediatric clinicians and researchers are often encountering several challenges when applying preventative and therapeutic programs and the characteristics of an optimal and comprehensive weight management strategy remain controversial [1,41]. Evidence suggests that the management of childhood obesity requires consideration of genetic, biological, behavioral, psychological, interpersonal, and environmental factors to induce sustainable lifestyle changes along with an in-depth understanding of these interactions to identify opportunities for intervention strategies [39].

### 3.1. Intervention Components

The most common preventative and therapeutic interventions applied and suggested by investigations are nutritional, PA, lifestyle, and educational methods. In adolescents with a high degree of obesity or advanced metabolic disease, clinical treatments including pharmacological and surgical strategies have also been suggested [20]. Due to the potential side effects of medical interventions, a careful evaluation and comparison of risks and benefits are necessary before implementing such interventions for pediatric patients with obesity [42]. It has been emphasized that pharmacotherapy and bariatric surgery should never be implemented in adolescents with obesity (and those with other vital untreated disorders) who have not engaged in healthy dietary and PA practices [20].

#### 3.1.1. Diet

It should be considered that nutritional approaches targeting childhood obesity are not limited to restricted energy intake but rather, the most appropriate nutrition strategy for long-term weight reduction and the promotion of metabolic and mental health is shifting to healthy food choices [43] that include predominantly whole food plant-based sources [44,45]. This dietary pattern restricts added sugars, refined grains, sweetened beverages, fast foods, calorie-dense snacks, and high-fat processed foods and includes fruits, vegetables, nuts, and whole-grains along with well-structured meal frequencies [6,44,46]. The weight-related benefits rising from plant-based diets are attributable to a reduced caloric intake and an increase in postprandial energy expenditure by a higher thermogenic response [47,48]. Additionally, whole food plant-based diets lead to favorable changes in cardio-metabolic and digestive health, which are both associated with further weight-related advantages as well [49,50]. Increasing nutritional literacy of children and their parents (regarding agriculture, food industries, food safety, cooking, and theoretical knowledge of energy balance, nutrition, and diets) could further promote sustainable changes that contribute to healthier dietary patterns [51]. While these are general principles and recommendations, no single diet should be prescribed or recommended as the best for all children with obesity [43]. Researchers believe that the optimal macronutrient composition depends on factors such as appetite, thermogenesis, energy homeostasis, and gut microbiota [52]. Furthermore, the ideal diet for treating overweight and obesity should be safe, efficacious, nutritionally adequate, culturally acceptable, and economically affordable [43]. To date, however, the majority of nutritional strategies targeting childhood obesity are still based on a “one-size-fits-all” model, which does not take into account the inter-individual variability [53], which often results in a reduced adherence rate to dietary changes [54].

#### 3.1.2. Physical Activity

On the other side of the energy balance equation, PA has been emphasized as a critical component for healthy body weight. Promoting PA is considered an effective intervention strategy in pediatric weight management [55,56], which attributes to the concept of energy flux [19]. Energy flux represents the rate of energy expenditure and energy intake [19,57], and a higher energy flux (obtained by increased PA) results in better regulation of energy balance during weight loss [57] and/or the prevention of weight gain [58]. PA could also result in favorable improvements in mental and physiological health, and both are indirectly associated with further weight-related advantages [59]. In children and adolescents with obesity, the most common barriers to engage in regular PA programs are lack of self-discipline, lack of someone to engage in PA with, self-consciousness about appearance [60], and decreased level of motivation due to the limited motor abilities and/or being out of shape [61]. Daily physical activities of children are not limited to regular physical education classes or sport/exercise engagements. Active travel to school, unstructured active play during school recess, and activities at home or playgrounds can be additional viable PA sources [32]. Currently, however, due to the COVID-19 pandemic and social lockdowns, the movement opportunities have been significantly diminished [62], and home exercises have been highly recommended [63].

#### 3.1.3. Lifestyle and Education

In addition to diet and PA, other lifestyle parameters (e.g., psychological behaviors, modifying sleep patterns) are considered effective interventions in weight management programs. Independently or along with educational interventions, additional lifestyle behaviors could further increase the efficiency of PA and dietary interventions [1]. A controlled experimental study showed that a two-year, multi-component obesity intervention focusing on a lifestyle educational curriculum resulted in beneficial changes in Body Mass Index (BMI) percentiles in the intervention groups [64]. There is also evidence suggesting that mindfulness interventions [65,66] and forethoughtfulness (defined as being oriented more towards the future than the present) [67] might be advantageous for improving obesity-related eating and behavioral patterns. Mindfulness, defined as the awareness that arises from purposefully paying attention in the present moment with non-judgment [68], is suggested as an effective intervention strategy targeting childhood obesity [69]. Evidence also indicates that sleep is an important modifiable risk factor for managing childhood obesity, as eating and PA behaviors can be affected by the quality and duration of sleep [70]. Modifying sleep patterns in school-age children resulted in healthy dietary patterns via decreased food consumption, in particular, thus promoting favorable weight outcomes [71].

In general, it has been well-established that multi-component interventions including PA, nutritional, lifestyle, and educational strategies have been shown to yield better outcomes than single-component strategies [1,41,72]. In a systematic review of the effectiveness of lifestyle interventions targeting children’s weight and cardio-metabolic health, beneficial outcomes were observed only following the multi-component interventions [73]. However, due to the complex interaction in these approaches, identifying the degree of effectiveness for each component remains controversial.

### 3.2. Intervention Settings

Almost all studies in the childhood obesity sector have critically investigated or discussed the environment where interventions are applied and the people who support and/or supervise weight management programs. It has been well-established that home, school, and community can all play important roles independently in shaping and stabilizing children’s lifelong health- and weight-related behaviors [74,75].

#### 3.2.1. Home

Parental beliefs, attitudes, behaviors, and social support are vital for a child’s health and body composition [76]. Available evidence indicates that parents are involved in about half of the interventions targeting childhood obesity, and successful improvements on children’s BMI are in 75% of studies with parental involvement [74]. Results show, from a meta-analysis of 22 randomized control trials examining home-based interventions to control childhood obesity, that parents can play a crucial role in managing children’s weight by facilitating, motivating, and coaching the healthy behaviors of their children [77]. Due to the close familiarity of parents and their child, parents may better understand and consider the lifestyle parameters contributing to the development of obesity in their child [78]. Although parent-only interventions may be more cost-effective compared to school- and community-based programs [79], evidence suggests to combine home-based programs with other settings to deliver more favorable effects on anthropometry and BMI in children [80]. The effectiveness of grandparental supervision, on the other hand, has been reported to be close to zero with no association between children’s BMI z-scores and grandparental child care (whether as the primary caregiver or co-residence) [81].

#### 3.2.2. School

School is an important setting for improving child health behaviors, as children spend a significant part of their daily life in schools [82,83]. Moreover, the following conditions of the school environment also benefit the setting for implementing overweight/obesity-related interventions: schools offer a structured environment for applying interventions with ease; schools may provide one or two meals per day and, therefore, potentially dictate healthy food choices in their cafeteria; schools usually provide opportunities for PA and active games during recess and daytime; schools produce and expand extracurricular educational resources and wellness policies for both children and parents; schools could run an indirect competitive and encouraging atmosphere to promote children’s motivation and adherence towards interventions; schools could introduce their physical education instructors and/or athletic trainers as role-models; schools benefit from the contribution of staff and teachers to facilitate, deliver, and supervise the interventions [74,84,85]. Interestingly, successful school-based interventions are also highly effective in improving children’s anti-obesogenic behaviors at home [83]. However, because of time and budget constraints, many schools are not able to implement health and weight management programs [72].

#### 3.2.3. Community and Clinics

In addition to home- and school-based strategies, the community environment and clinical settings are also common areas for managing childhood obesity and prevention [20,75]. Community interventions targeting obesity incorporate policies and strategies and aim to reduce the population risk of obesity [75]. These interventions involve but are not limited to the availability and use of health and fitness facilities, media-based activities, and health-oriented businesses by local and central administrations [1,86]. The EPODE program (Ensemble Prévenons l’Obésité Des Enfants: together, let us prevent childhood obesity) could be considered a successful example of a community-based intervention, which emphasizes a multifactorial approach targeting childhood obesity at different community levels [87]. Interventions using a community-based approach could achieve the long-term goals of reducing the prevalence of childhood obesity [88], especially for children who live in low-income societies [75]. Clinical or primary-care interventions, on the other hand, include any medical or non-medical strategies implemented by healthcare and pediatric specialists [89]. Clinical and community-level interventions can significantly improve lifestyle patterns when applied simultaneously [90]. Significant improvements in body weight have been achieved in pre-school children aged 2–5 years following multi-component clinical interventions (e.g., PA, nutrition, education) with parental involvement [91]. However, reports from different meta-analytic studies indicate poor effectiveness of primary-care programs on childhood obesity [89,92,93], which might be attributed to a dose-response relationship, where the frequency and duration of treatment contact highly affect the outcomes [1]. Additionally, the “sustainability” of intervention effects could be another limitation in clinical approaches, as the time of engagement is limited compared to other settings [94,95].

In general, it seems that due to the multi-factorial nature of childhood obesity, a maximally efficient strategy to manage childhood obesity requires integrating multiple settings for delivering multi-component interventions. Evidence shows that school-based interventions with family inclusion have the largest effect on weight outcomes when multi-component programs are implemented, including PA and diet [84,96]. Results from a study comparing the effectiveness of home versus school settings on nutritional habits, PA behaviors, and BMI changes showed that the home environment had a stronger association with health in general compared to the school setting [97]. It should be mentioned, however, that parental involvement in many preventive studies can be more effective in pre-school and early-school children, whereas school- and community-based intervention strategies lead to more favorable outcomes in older children and adolescents [1], particularly for those who are above twelve [98]. Nevertheless, to enhance the effectiveness of strategies, it appears important that parents permanently engage in supporting and reinforcing their children’s health behaviors [99].

Some limitations can affect the progress of weight management programs, similar to any preventative and therapeutic strategy. Time and financial resources are major limitations for the implementation of multi-component weight management strategies [100]. In addition, poor awareness and lack of self-discipline have been reported as personal barriers when adhering to a healthy lifestyle [60]. Furthermore, difficult-to-reach goals set by parents and clinicians are considered a vital but hidden limitation, as it is well-established that strict targets may often lead to failure in weight control programs in children [101]. Age also appears to be an important moderator for weight control outcomes as older children displayed larger and more beneficial effects than younger children following weight-management interventions [92,93]. Beyond these limitations, the obesity prevention strategies seem to follow a dose-effective manner, as more intensive and longer-lasting interventions are associated with better outcomes in children [93]. Further, it appears that purposeless and/or unsupervised strategies not based on the individual needs and personal characteristics of the targeted child could minimize intervention effects [54].

## 4. Personalized Strategies

**Figure 1:**
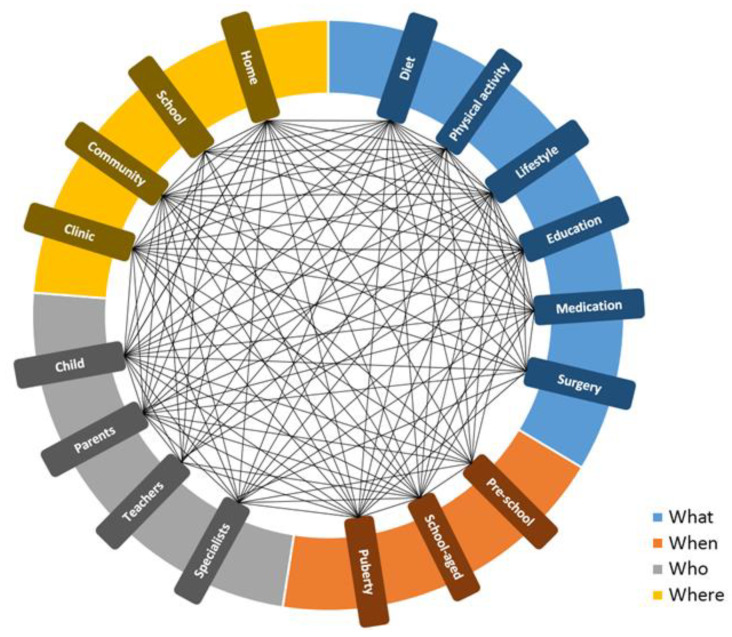
Conceptual 4W model describing the complexity of interactions between research-based modules contributing to the management of childhood obesity.

Despite a wealth of scientific information on a wide range of interventions and strategies targeting childhood obesity, the translation and transfer of this knowledge into a practical approach seem highly challenging. According to a comprehensive study by the Institute of Medicine (IOM), which analyzed more than 800 scientific reports, the progress of obesity prevention was not favorable in the national trend data, and data was not translatable into clearly scalable strategies [102,103]. It has been reported that meta-analytic approaches for identifying solutions to obesity, which is a complex health problem, could not deliver favorable practical information [102]. As a result of obesity’s complexity, the condition seems not only limited to its etiology, but also to intervention strategies targeting childhood obesity. Given the interaction between various components of preventative/treatment approaches, the management of childhood obesity remains highly complex. Figure 1 shows a conceptual model that describes the complexity of interactions between key aspects of four research-derived categories (“What”, “When”, “Who”, and “Where”), which are critical in the management of childhood obesity. “What” refers to the components including diet, PA, other lifestyle interventions, education, medication, and surgery. “When” stands for different age groups that are targeted (including pre-school, school-age, and puberty). “Who” represents the involved population such as the child, parents, teachers, and specialists. Finally, “Where” appoints different settings including home, school, community, and clinic (Figure 1).

Recently, the implementation of personalized dietary approaches to managing complicated health problems (e.g., cardiovascular and metabolic disorders) has been increasing [104,105]. Personalized interventions could be defined as advanced and detailed models of clinical/primary care interventions. Given the available data, current clinical interventions seem to have some limitations. Clinical strategies focus primarily on treatment rather than prevention and thus are often conducted in close coordination with the primary healthcare system with high accessibility and frequency of visits mostly in the clinical setting [106]. Clinical approaches mainly focus on nutritional and medical interventions with lower attention on lifestyle, educational, and movement strategies [20]. Lifestyle counseling, including suggestions for PA by health specialists, remains below an acceptable level [107,108,109] even though the importance of lifestyle interventions by physicians and/or health care providers has been well-documented in patients’ health- and weight-related behaviors [107,108]. This may be attributed to inadequate knowledge and training, office time constraints, and poor personal habits of specialists/physicians [107,109]. Rather than providing general information, a personalized strategy uses a broad range of info on individual characteristics to develop targeted nutritional and non-nutritional advice, products, or services assisting people to reach their goals via a purposeful approach based on their current behaviors, preferences, barriers, and objectives.

Personalized dietary approaches have been reported previously as a promising topic of research in the treatment of obesity [110]. To date, evidence supporting personalized strategies to manage obesity has come from clinical and observational studies mostly in the area of nutrition. The Academy of Nutrition and Dietetics developed the personalized nutritional approach NCP (Nutrition Care Process) based on nutritional assessment, diagnosis, planning, and monitoring. This multi-step model was designed to structure individualized nutritional care targeting childhood obesity and was effective in different investigations [22,111,112]. In a review study assessing the effects of NCP-based educational programs (including education on meal planning, portion control, healthy snack selection, and cooking with plant-based sources), favorable outcomes were reported regarding childhood obesity [113].

In addition to nutritional interventions, the limited available data supports the effectiveness of other personalized interventions on weight and/or health outcomes in children and adolescents. A 3-month personalized PA intervention using an internet-based program showed significant effects on psychosocial health and PA level in adolescents [114]. Evidence consistently indicates that when compared to generalized programs, personalized technology-based PA interventions are more effective at modifying health behaviors [115]. Results from a study on adolescents with obesity or diabetes show that 16 weeks of personalized exercise (based upon baseline fitness level of participants)—with parental support and ongoing motivation—can improve PA level and result in a sense of personal health [116]. Additionally, a controlled experimental study showed beneficial effects of personalized lifestyle coaching on childhood obesity [117], in which a health coach called child-parent pairs separately by telephone for a total of 21 sessions. There is also evidence indicating children’s health behaviors, particularly sleep patterns, could be improved following personalized educational interventions for mothers with 3- to 5-year-old children [118].

In general, personalized recommendations on the personal needs of a child and his/her family could be a promising approach for the prevention and treatment of obesity. A comprehensive personalized approach targeting childhood obesity may include, among others, nutritional, educational, and PA-based intervention strategies at various settings to alter lifestyle patterns and attitudes. The overall consensus is that implementing a well-proposed personalized program not only maximizes desirable outcomes but also contributes to the sustainable adherence of a healthy lifestyle pattern [54]. In addition, due to the purposeful nature of personalized interventions, time and budget could be partially saved following this approach. Similar to other successful programs, a personalized program should further combine education and motivation to obtain slow but sustainable weight and health benefits [43].

## 5. EPISTCO Model

**Figure 2:**
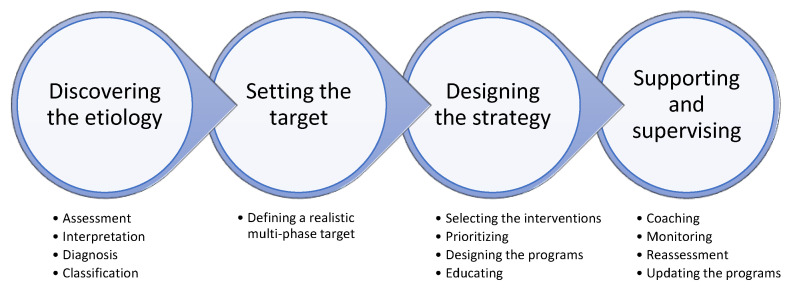
Schematic design of EPISTCO (Etiology-Based Personalized Intervention Strategy Targeting Childhood Obesity) model, which is based on four multi-stage steps.

Considering the complicated facts in the etiology and management of childhood obesity and in order to establish a novel translational link between the literature and practice, this narrative review presents a basis for an Etiology-Based Personalized Intervention Strategy Targeting Childhood Obesity (EPISTCO) (Figure 2), to provide a framework for the purposeful prevention and treatment of childhood obesity.

The EPISTCO model highlights that the design of a personalized program targeting childhood obesity requires an understanding of the complex etiology of excess weight gain by assessing a series of biological, nutritional, behavioral, and environmental factors. Unlike previously described models, the EPISTCO model implements a multi-component intervention program within multiple settings and considers personalized priorities for the components and settings. In this structured multi-disciplinary and etiology-based approach, the programs are highly adaptable based on individual and environmental barriers and potentials. 

The EPISTCO model includes four multi-stage steps (Figure 2). The first and probably the most important step is “discovering the etiology of obesity”, which most likely requires a clinical setting. This step consists of four stages including (a) assessments, (b) interpretation of data, (c) diagnosis of the relevant causes, and (d) classification of the causes. Assessments (e.g., physical characteristics, eating habits and disorders, sleep patterns, etc.) can be made via questionnaires, field tests, and laboratory measurements and are depending on the availability of time, equipment, and specialists. Table 1 represents the most important assessment items summarized in nine general categories that provide viable information to design an EPISTCO.

Step 2 is “setting the target”, in which a multi-phase goal is defined according to the information obtained from Step 1. The emphasis is to set a realistic target, as difficult-to-reach targets often lead to failure. Step 3, “designing the strategy”, consists of the following stages: (a) selecting the most appropriate interventions, (b) prioritizing interventions according to stage “d” from Step 1, (c) designing program schedules along with extra general recommendations, and (d) educating both the child and parents about the next step in conjugation with motivational incentives. At the end of Step 3, the next visit(s) must be set according to a time-based, target-based, or problem-based style. Accordingly, this step will also determine the role of different settings including home, schools and communities. Finally, Step 4, “supervising and supporting”, consists of three stages: (a) individualized direct or indirect coaching and psychological supporting, (b) monitoring, (c) reassessment, analysis of the progress as well as evaluating problems, and (d) revising and updating the program. This step returns most likely to the clinical setting to connect Step 4 and Step 1. Rather than circling back, the intention is to continue the procedure to stabilize health behaviors.

To further enhance the understanding of the characteristics of this personalized approach, the following examples can be considered. For a child with obesity who has a proper quantity and quality of nutrition, interventions should prioritize PA and other lifestyle patterns. A more active child with excess body weight, on the other hand, with other causes (e.g., unhealthy food choices, sleep patterns, lifestyle, biochemistry) may require a different approach that should be scrutinized during initial assessments in order to design and suggest a purposeful etiology-based program. To reach favorable results, it is, nevertheless, highly recommended that all steps and stages of the EPISTCO approach are conducted and supervised by well-experienced pediatric specialists in different sub-disciplines of health. Moreover, every stage throughout the process should be well documented. The gathered data will also provide viable information that enhances the understanding of the etiology of obesity, which is critical for the improvement of the effectiveness of such personalized approaches.

## 6. Conclusions

The high prevalence of childhood obesity is a major threat to future public health and available literature indicates that weight-related nutritional, PA, and lifestyle recommendations are not met by the majority of children. Given the complex and multi-factorial nature of obesity in both etiology and management, it appears that there is a fundamental need to develop and apply personalized approaches to prevent and treat childhood obesity. As a practical, purposeful, and promising suggestion, the EPISTCO model emphasizes incorporating various approaches, including nutritional, lifestyle, and PA, that are prioritized, prescribed, and supervised based on the individual needs and personal characteristics within multiple settings.

In general, the EPISTCO model offers a purposeful framework for pediatric researchers and specialists that contributes to a better understanding of the interplay between various factors associated with childhood obesity, which can increase the efficacy of interventions. While it includes a comprehensive approach towards minimizing childhood obesity, not all aspects need to be implemented in every situation.

## Figures and Tables

**Table 1:** Essential prerequisite information to design and conduct an etiology-based personalized intervention strategy targeting childhood obesity.

	Questionnaire	Laboratory Tests	Field Tests
1. Individual and parental information	*		
2. Self-reported targets	*		
3. Facilities/limitations in personal environment	*		
4. Energy balance status with a short history	*	*	
5. Lifestyle behaviors with a short history	*		
6. Body composition status with a short history	*	*	
7. Clinical status with a short history	*	*	
8. Biochemistry status with a short history		*	
9. Physical fitness status with a short history		*	*

## Data Availability

No new data were created or analyzed in this study. Data sharing is not applicable to this article.
